# Supplementary material for: Bacterial Production of CDKL5 Catalytic Domain: Insights in Aggregation, Internal Translation and Phosphorylation Patterns
Source: Int J Mol Sci. 2024 Aug 15;25(16):8891. doi: 10.3390/ijms25168891 (PMC11354467; doi:10.3390/ijms25168891)
Supplement: Supplementary file 1 [file ijms-25-08891-s001.zip › ijms-3089955-supplementary.pdf]

Supplementary materials to

Bacterial Production of CDKL5 Catalytic Domain: Insights in Aggregation, Internal Translation and Phosphorylation Patterns

Andrea Colarusso [1,†], Concetta Lauro [1], Luisa Canè [2,3], Flora Cozzolino [1,2] and Maria Luisa Tutino [1,4,*]

[1] Department of Chemical Sciences, University of Naples Federico II, Complesso Universitario Monte S. Angelo, Via Cintia 4, 80126 Naples, Italy; and.colarusso@gmail.com (A.C.); concetta.lauro@unina.it (C.L.); flora.cozzolino@unina.it (F.C.)

[2] CEINGE Advanced Biotechnologies, Via G. Salvatore 486, 80145 Naples, Italy; canel@ceinge.unina.it

[3] Department of Translational Medical Sciences, University of Naples Federico II, Via Sergio Pansini 5, 80131 Naples, Italy

[4] Istituto Nazionale Biostrutture e Biosistemi I.N.B.B., Viale Medaglie D'Oro 305, 00136 Roma, Italy

* Correspondence: tutino@unina.it; Tel.: +39-081674317

† Current address: Department of Integrative Structural and Computational Biology, The Scripps Research Institute, 9060 Activity Rd Unit F, San Diego, CA 92126, USA.

Index

S1. Evaluation of the enzymatic activity of CDKL5ΔC isolated from either soluble or insoluble cellular fractions

S2. Expression of Sumo- and GST-tagged CDKL5ΔC constructs with different truncations

S3. Co-expression of Sumo-tagged CDKL5ΔC constructs with different truncations with chaperones at 15 °C

S4. Co-expression of Sumo-tagged CDKL5ΔC with chaperones at 25 °C

S5. Co-expression of untagged CDKL5ΔC with chaperones

S6. Autophosphorylation activity of CDKL5ΔC purified from *E. coli* and insect cells

S7. Setup of a quantitative procedure to determine Y171 phosphorylation levels in different CDKL5ΔC fragments produced in *E. coli* and insect cells

S8. IMAC on BL21(DE3) soluble lysates producing H6-Ph_Sumo-CDKL5(1-352)

S9. Isolation of Hsp60 in absence of soluble CDKL5ΔC

Table S1. Composition of buffers used to solubilize CDKL5ΔC Ibs

Table S2. List of primers used in this work

S1. Evaluation of the enzymatic activity of CDKL5ΔC isolated from either soluble or insoluble cellular fractions

As Ec_Tat-CDKL5(1-352)-His is totally insoluble when expressed in *E. coli* strains (Figure 3), we tried its purification and refolding from inclusion bodies (Ibs) collected from BL21(DE3) lysates after expression at 15 °C. CDKL5ΔC Ibs proved to be nonclassical Ibs because they could be easily solubilized with mild methods, including sonication, the addition of low concentrations of ionic detergents, and extreme basic pH. Table S1 reports some of the buffers that allowed the solubilization of most CDKL5ΔC Ibs.

After solubilization in buffers A-E, CDKL5ΔC preparations were dropwise diluted in a refolding buffer. All such preparations contained soluble protein (Figure S1A). To measure the enzymatic activity of refolded Ec_Tat-CDKL5(1-352)-His, two assays were used. First, we analyzed the phosphorylation levels of Y171 which is recognized as a typical autophosphorylation site of CDKL5, by using a Western blot with an affinity purified anti-CDKL5 pY171 antibody [1]. In our autophosphorylation assay, an anti-His Western blot confirmed the presence of the target proteins (Figure S1B, left panel), but their anti-CDKL5 pY171 profile was the same as the one of the kinase-dead (KD) mutant (NC in Figure S1B, right panel) that is known to be catalytically inactive [2]. On the other hand, the phosphorylation was clearly distinguishable in a Ph_Sumo-Tat-CDKL5(1-352)-His purified in native condition (PC in Figure S1B). This result was confirmed by an in vitro kinase assay involving the use of EB2 as a substrate, a known phosphorylation target of CDKL5 [1,3]. While Ph_Sumo-Tat-CDKL5(1-352)-His could phosphorylate its substrate (PC in Figure S1C, right panel), all the proteins deriving from solubilization in A-E buffers were almost totally inactive. Attempts to refold the solubilized Ec_Tat-CDKL5(1-352)-His with other approaches (e.g., dialysis, fast dilution, removal of detergents with polystyrene beads) failed (data not shown). Based on these results, further studies focused on the isolation of CDKL5ΔC variants only in native conditions from cellular soluble fractions.

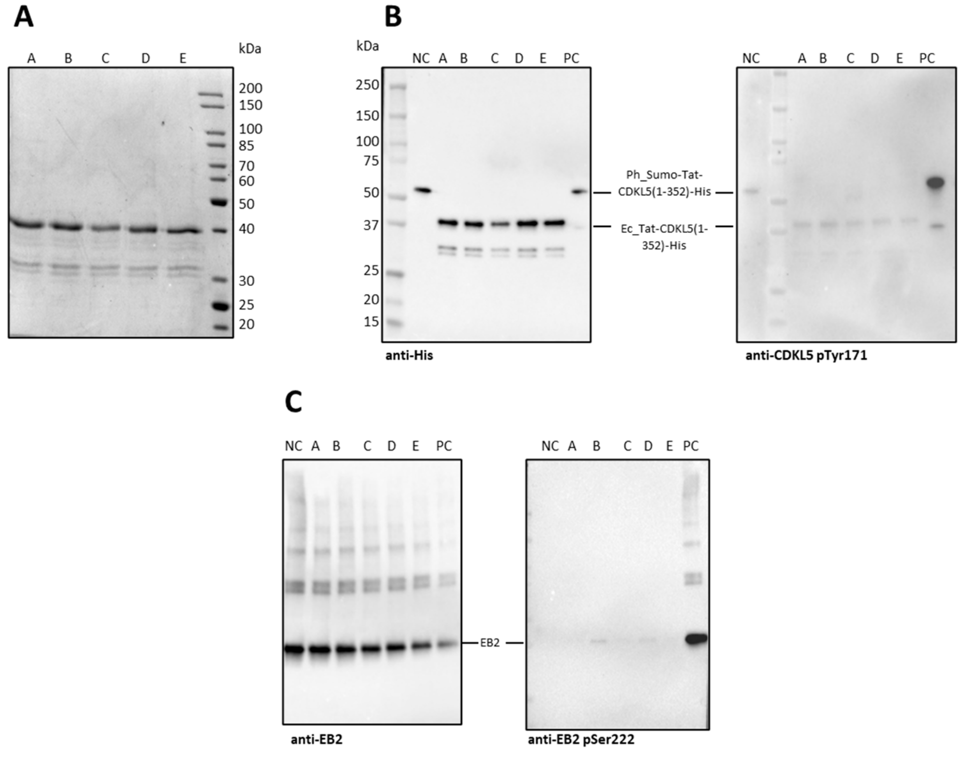

Figure S1 Activity of Ec_Tat-CDKL5(1-352)-His preparations after Ibs solubilization. (**A**) Soluble protein profiles of Ec_Tat-CDKL5(1-352)-His after solubilization in buffers A-E (Table 1) and dropwise refolding. (**B**) CDKL5 pY171 levels of refolded Ibs. The left panel is representative of total proteins detected with an anti-His Western blot. The right panel is representative of phosphorylated CDKL5ΔC revealed by an anti-CDKL5 pTyr 171 antibody. (**C**) CDKLΔC activity on EB2. The left panel represents total EB2 revealed by an anti-EB2 antibody, while the right panel is a Western blot of phosphorylated EB2 highlighted by an anti-EB2 pSer222 antibody. Lanes A-E indicate in which buffers CDKL5ΔC were solubilized (Table S1). NC: Ph_Sumo-Tat-CDKL5(1-352)-His M10V_KD; PC: Ph_Sumo-Tat-CDKL5(1-352)-His M10V.

S2. Expression of Sumo- and GST-tagged CDKL5ΔC constructs with different truncations

CDKL5ΔC constructs were produced in *E. coli* BL21(DE3) with truncations at the level of 303, 352, and 498 residues with either a GST or Sumo tag. Furthermore, the GST-tagged constructs were produced both as wt versions and M10V mutants, while the Sumo-tagged variants were only M10V mutants. Figure S2A shows that all Ph_Sumo-tagged constructs are mainly insoluble regardless of the chosen C-terminal truncation site. Similarly, Figure S2B highlights that all GST-tagged constructs are mostly insoluble and that the wild-type proteins always generate a further fragmentation product due to an internal translation starting at the level of M10 (red asterisks in Figure S2B). Figure S2C shows that even if the three Sumo-tagged constructs have similar levels of solubility, Ph_Sumo-Tat-CDKL5(1-303)-His M10V almost totally lost its C-terminal His-tag as highlighted by the anti-His Western blot (right panel). Figure S2D shows the fragmentation pattern of Ph_Sumo-Tat-CDKL5(1-498)-His M10V, GST-Tat-CDKL5(1-498) wt, and GST-Tat-CDKL5(1-498) M10V (lanes 2, 3, and 4, respectively), in soluble lysates from recombinant BL21(DE3), highlighting that the GST constructs are further fragmented in comparison with the Sumo-tagged variant.

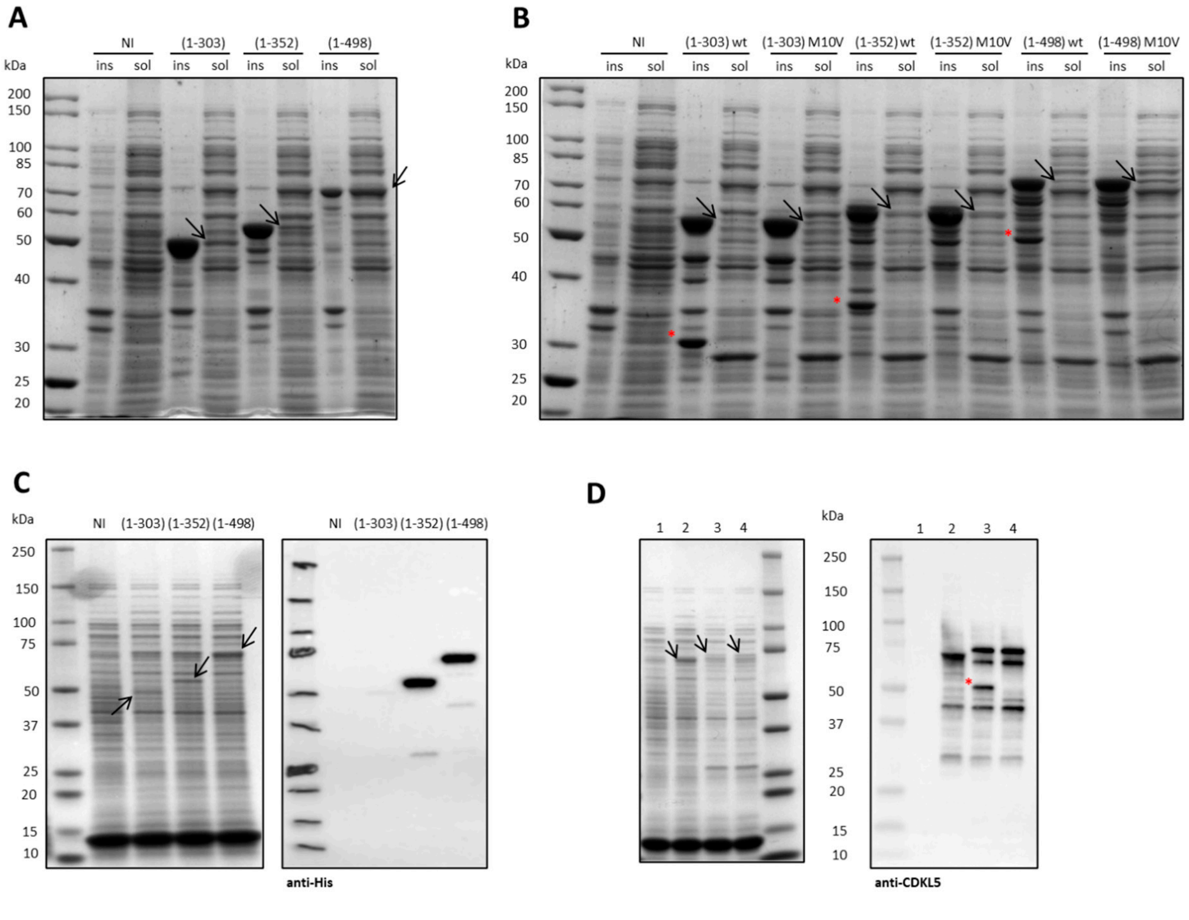

Figure S2 Production of CDKL5ΔC variants with either Sumo or GST tags. (**A**) SDS-PAGE profile of insoluble (ins) and soluble extracts (sol) of BL21(DE3) either not induced (NI), producing Ph_Sumo-Tat-CDKL5(1-303)-His M10V (1-303), Ph_Sumo-Tat-CDKL5(1-352)-His M10V (1-352), Ph_Sumo-Tat-CDKL5(1-498)-His M10V (1-498). (**B**) SDS-PAGE profile of insoluble (ins) and soluble extracts (sol) of BL21(DE3) either not induced (NI), producing GST-Tat-CDKL5(1-303) either as wt (1-303 wt) or M10V mutant (1-303 M10V), GST-Tat-CDKL5(1-352) either as wt (1-352 wt) or M10V mutant (1-352 M10V), GST-Tat-CDKL5(1-498) either as wt (1-498 wt) or M10V mutant (1-498 M10V). (**C**) SDS-PAGE (left panel) and anti-His Western blot (right panel) of soluble extracts from non-induced cells, BL21(DE3) producing Ph_Sumo-Tat-CDKL5(1-303)-His M10V (1-303), Ph_Sumo-Tat-CDKL5(1-352)-His M10V (1-352), and Ph_Sumo-Tat-CDKL5(1-498)-His M10V (1-498). (**D**) SDS-PAGE (left panel) and anti-CDKL5 Western blot (right panel) of soluble extracts from BL21(DE3) either not induced (lane 1), producing Ph_Sumo-Tat-CDKL5(1-498)-His M10V (lane 2), GST-Tat-CDKL5(1-498) wt (lane 3), and GST-Tat-CDKL5(1-498) M10V. Black arrows indicate the full-length proteins in soluble extracts, while the red asterisks highlight the N-terminally truncated forms of wild-type products due to the internal translation start.

S3. Co-expression of Sumo-tagged CDKL5ΔC constructs with different truncations with chaperones at 15 °C

Differently truncated Sumo-tagged CDKL5ΔC variants were co-expressed with *E. coli* chaperones at 15 °C and the soluble extracts were analyzed. The solubility levels of each variant were increased with different combinations of chaperones regardless of the truncation point as visible by SDS-PAGE analysis (Figure S3A). However, the Western blot analysis further confirmed the loss of the C-terminal His tag in the case of the 1-303 construct (Figure S3B).

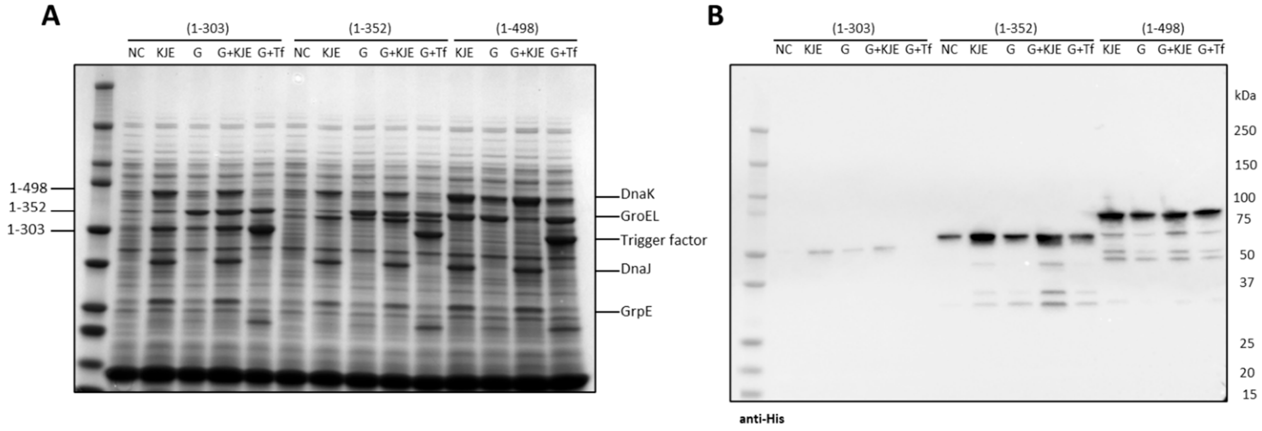

Figure S3 Production of Sumo-tagged CDKL5ΔC variants with different *E. coli* chaperones at 15 °C. SDS-PAGE (**A**) and anti-His Western blot analysis (**B**) of BL21(DE3) soluble extracts after co-expression of the target proteins with chaperones. CN, CDKL5ΔC alone; KJE, DnaK + DnaJ + GrpE; G, GroELS; G + KJE, GroELS + DnaK + DnaJ + GrpE; G + Tf, GroELS + trigger factor. The number ranges on the left indicate the encompassed CDKL5 sequence included in each construct.

S4. Co-expression of Sumo-tagged CDKL5ΔC with chaperones at 25 °C

Given that the *E. coli* chaperones are likely to work better at higher temperatures, their co-expression with Ph_Sumo-Tat-CDKL5(1-352)-H8 M10V at 25 °C was attempted. However, as shown in Figure S4, the co-expression with chaperones at 25 °C (lanes 2-6) triggered lower solubility than 15 °C (lanes 7-8).

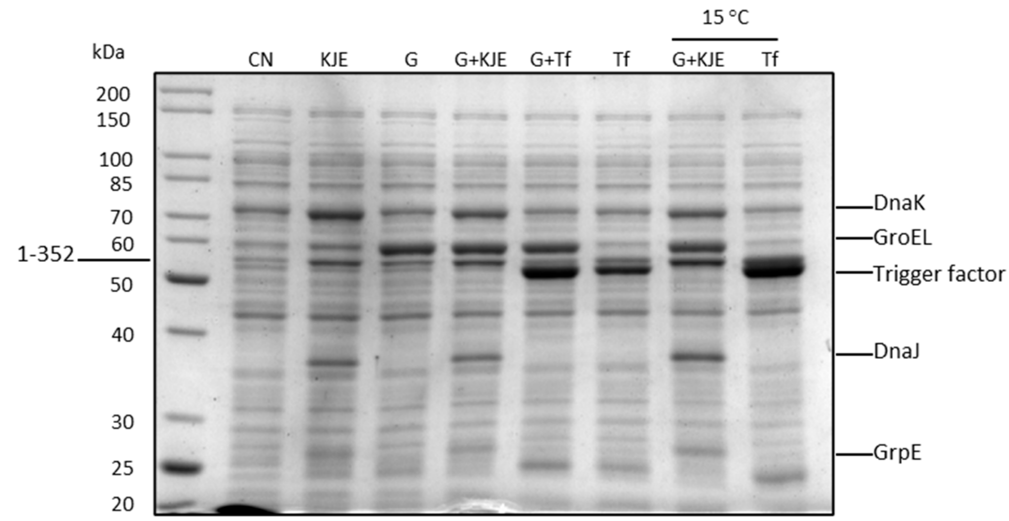

Figure S4 Production of Sumo-Tat-CDKL5(1-352)-H8 M10V with different *E. coli* chaperones at 25 °C. SDS-PAGE of BL21(DE3) soluble extracts after co-expression of the target protein with chaperones. CN, CDKL5(1-352) alone; KJE, DnaK + DnaJ + GrpE; G, GroELS; G + KJE, GroELS + DnaK + DnaJ + GrpE; G + Tf, GroELS + trigger factor; Tf, trigger factor. The number ranges on the left indicate the encompassed CDKL5 sequence included in each construct.

S5. Co-expression of untagged CDKL5ΔC with chaperones

To test whether in the presence of chaperones the tagging with Sumo is unnecessary to achieve soluble CDKL5ΔC, we generated Ph_CDKL5(1-352)-H8 M10V, which is devoid of both Sumo and Tat and triggered its production at 15 °C together with different combination of chaperones. Although the protein production was visible in the total lysate (lane 8 in Figure S5), it was totally insoluble in the absence of chaperones (lane 2) and slightly soluble in the presence of chaperones (lanes 3-7). However, the presence of the Sumo tag guaranteed a considerably higher solubility (lane 9).

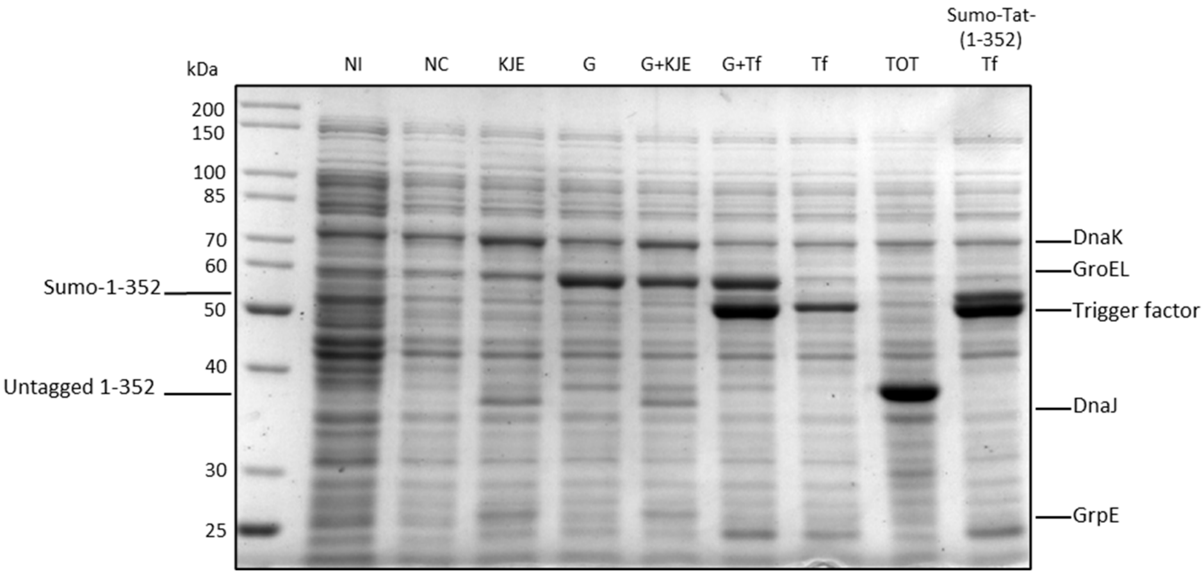

Figure S5 Production of CDKL5(1-352)-H8 M10V with different *E. coli* chaperones at 15 °C. SDS-PAGE of BL21(DE3) soluble extracts after co-expression of the target protein with chaperones. NI, not induced; NC, CDKL5(1-352) alone; KJE, DnaK + DnaJ + GrpE; G, GroELS; G + KJE, DnaK + DnaJ + GrpE + GroELS; G + Tf, GroELS + trigger factor; Tf, trigger factor; TOT, CDKL5(1-352) whole lysate. The number ranges on the left indicate the encompassed CDKL5 sequence included in each construct.

S6. Autophosphorylation activity of CDKL5ΔC purified from *E. coli* and insect cells

The capability of recombinant CDKL5ΔC to either *cis*- or *trans*-phosphorylate T169 and Y171 in its activation loop was tested. Particularly, to highlight whether the used cell factory for CDKL5ΔC production influences its phosphorylation levels, recombinant proteins collected from either *E. coli* or insect Sf9 cells were compared. Sumo-tagged catalytically active CDKL5(1-498) was purified from *E. coli* BL21(DE3) soluble extracts as reported in the main text, while GST-tagged CDKL5(1-498) from Sf9 was purchased (#ab131695, Abcam). Such preparations were incubated with MgATP either alone or mixed with a shorter kinase-dead mutant (Ph_Sumo-Tat-CDKL5(1-352)-His M10V_KD).

As visible from the SDS-PAGE gel in Figure S6, similar amounts of proteins were used in each assay (top panel). However, the commercial CDKL5(1-498) from Sf9 was heavily proteolyzed (last six lanes in Figure S6). Nevertheless, the insect-derived enzyme was the only one that showed autophosphorylation ability in this experiment. Furthermore, both the single pTyr171 (middle panel) and double pThr169/pTyr171 signals (bottom panel) increased over time after incubation with MgATP of the commercial enzyme. However, no trans-phosphorylation of the KD protein could be observed. Although the *E. coli*-derived protein seemed not phosphorylated, its phosphorylation signals were clearly distinguishable in other experiments (Figure 9 in the main text). This indicates that the phosphorylation levels of the two proteins are so different that the ones relative to the bacterially synthesized CDKL5(1-498) are obscured by the eukaryotic one.

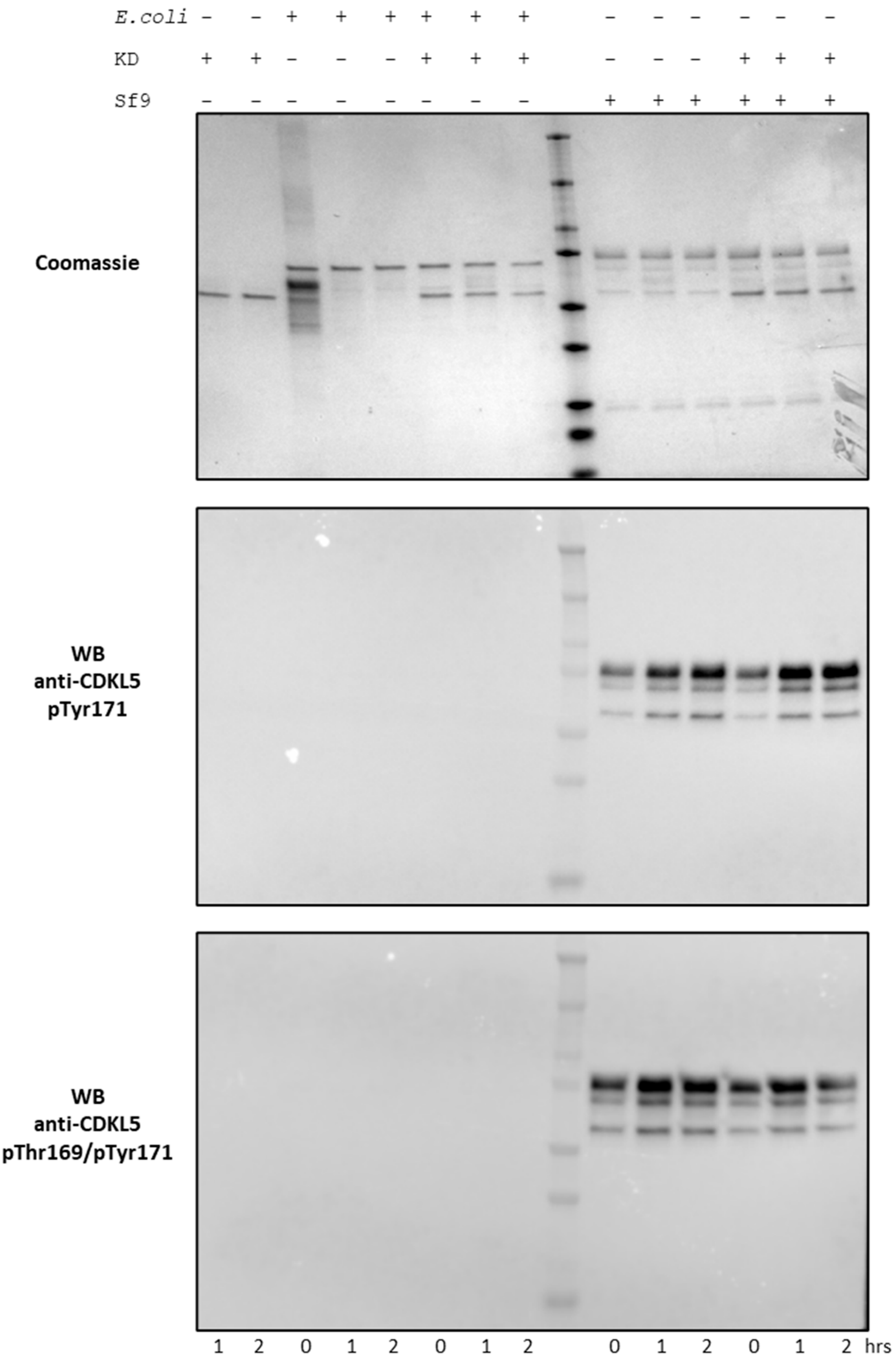

Figure S6 Autophosphorylation of T169 and Y171 in CDKL5ΔC from *E. coli* and insect cells. A catalytically active CDKL5 variant spanning 1-489 residues, purified from either *E. coli* or Sf9 insect cells, and a shorter catalytically inactive CDKL5 variant (Ph_Sumo-Tat-CDKL5(1-352)-His M10V_KD) were incubated either together or alone with ATP at 30 °C for 2 h. Representative samples were analyzed at increasing time points via SDS-PAGE (upper panel), anti-CDKL5 pTyr171 Western blot (middle panel), and anti-CDKL5 pThr169/pTyr171 (bottom panel).

S7. Setup of a quantitative procedure to determine Y171 phosphorylation levels in different CDKL5ΔC fragments produced in *E. coli* and insect cells.

The purified catalytically active CDKL5 variant (Ph_Sumo-Tat-CDKL5 (1-352)-His WT), the corresponding kinase-dead mutant (Ph_Sumo-Tat-CDKL5 (1-352)-His M10V_KD) both produced in *E. coli* cells, and the commercial CDKL5ΔC variant (1-498) purified from Sf9 insect cells (#ab131695, Abcam) were resuspended in Laemmli buffer and fractionated by SDS PAGE. The protein bands stained by colloidal coomassie at the expected molecular weights for the two constructs and the protein from insect cells were excised from the gel and in situ hydrolyzed by trypsin. The peptide mixtures were analyzed via LC-MS/MS (Figure S7.1 and S7.2) on a high-resolution mass spectrometer equipped with an Orbitrap analyzer. The post-translational modifications (i.e., phosphorylation) were mapped using the MASCOT software. Additionally, the relative abundances (percentage) of phosphorylated peptides were calculated by measuring the area of the correspondent chromatographic peak (extracted ion current approach) in comparison with the total area of peaks associated with modified and unmodified peptides [4,5]. According to the fragmentation spectra (Figure S7.1 panel D and Figure S7.2 panel D), the MASCOT software suggested that only in the protein samples corresponding to the active CDKL5 variants (produced in either *E. coli* or insect cells, respectively), the tryptic peptide 159-175 (NLSEGNNANY**T**E**Y**VATR) including the activation loop and the potential phosphorylation sites T169 and Y171 (in bold) was partially mono-phosphorylated and that the post-translational modification occurred exclusively on Y171. The same tryptic peptide 159-175 from the kinase-dead mutant produced in *E. coli* cells was not phosphorylated (data not shown).

The doubly phosphorylated species on both T169 and Y171 were not detected either in the bacteria or the insect-expressed protein. The chromatographic areas of peaks corresponding to the phosphorylated and no-phosphorylated peptide ions were then measured from LC traces of the tryptic mixture of CDKL5ΔC deriving from the protein expressed in bacteria (Figure S7.1 panels B and C) or in insect cells (Figure S7.2 panels B and C).

The percentage (%) of the phosphorylated peptide 159-175 on Y171 in both samples, was calculated according to the formula shown below:

$$\frac{\text{area of phosphorylated peptide ion peak}}{\text{area (phosphorylated + no/phosphorylated) peptides}} \text{x100}$$

Formula for calculating the phosphorylation percentage of peptide 159-175

In total, 0.8% of CDKL5 fragment molecules produced in *E. coli* resulted in having a phosphorylated Y171, while 20% of the CDKL5ΔC fragments produced in Sf9 cells were phosphorylated on the same residue, a result in good agreement with the intensity of Western blot signals.

The absence of phosphorylation on T169 was further assessed by a subdigestion with endoproteinase Glu-C which, by hydrolyzing at the C-terminus of glutamic acid (E) residues, generated two peptides: 159-170 and 171-175. The molecular weight of parte ion (experimental and theoretical m/z 1325.5528 and 1325.5531 Da) and the fragmentation spectra of peptides 159-170 confirmed the absence of modification on T169 (data not shown).

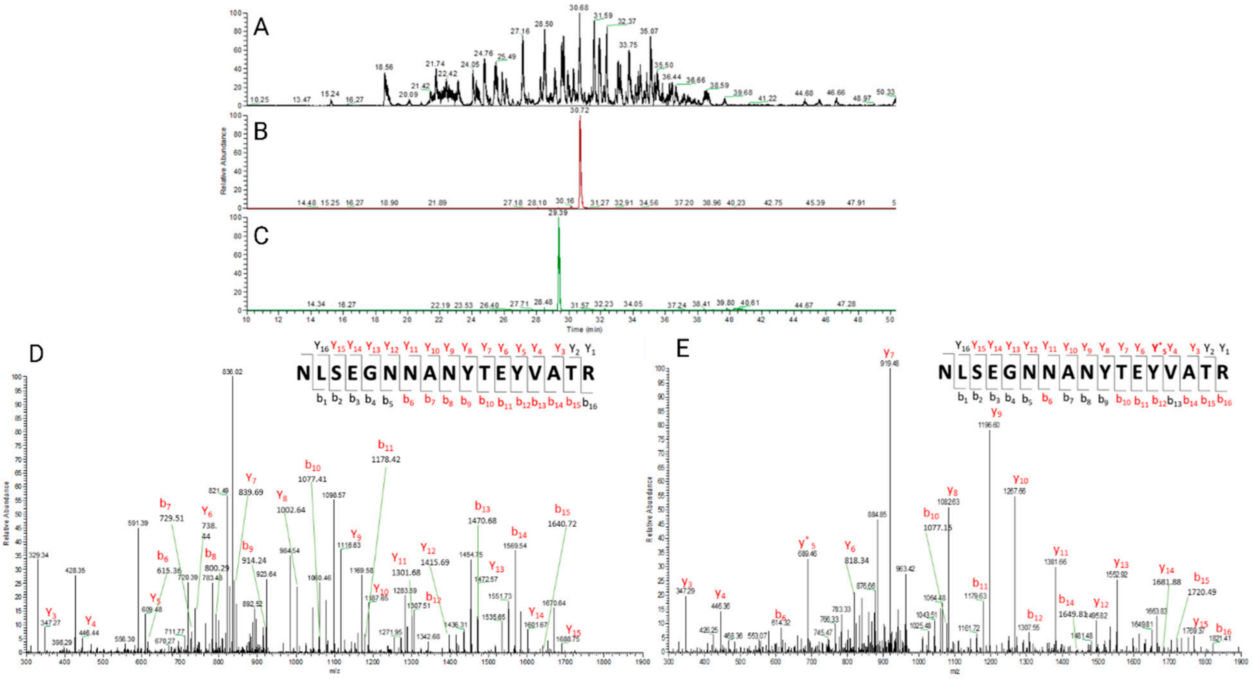

Figure S7.1: **LCMSMS analysis of tryptic mixture from CDKL5ΔC expressed in *E. coli.*** (A) LCMSMS profile of total tryptic mixture of CDKL5ΔC; (B) extracted ion chromatogram (XIC) of doubly charged 958.4481 m/z corresponding to the not phosphorylated peptide ion 159-175; (C) extracted ion chromatogram (XIC) of doubly charged 998.4343 m/z corresponding to the phosphorylated peptide ion 159-175; (D) fragmentation spectra of no phosphorylated (E) and phosphorylated 159-175 peptide. The peptide sequence is reported upper in the figure with its interpretation; the asterisk indicates the signal corresponding to the phosphorylated tyrosine.

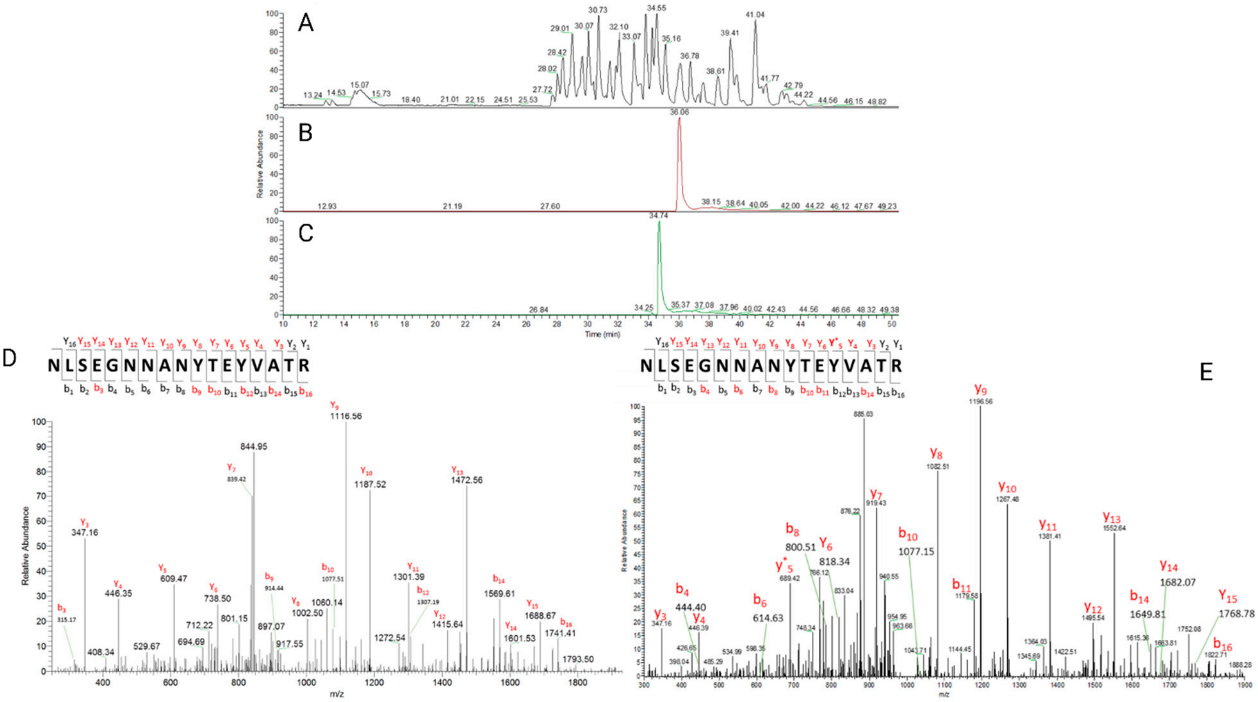

Figure S7.2: **LCMSMS analysis of tryptic mixture from CDKL5ΔC expressed in insect cells** (Baculovirus-infected SF9 cells)**.** (A) LCMSMS profile of total tryptic mixture of CDKL5ΔC; (B) extracted ion chromatogram (XIC) of doubly charged 958.4481 m/z corresponding to the not phosphorylated peptide ion 159-175; (C) extracted ion chromatogram (XIC) of doubly charged 998.4343 m/z corresponding to the phosphorylated peptide ion 159-175; (D) fragmentation spectra of no phosphorylated (E) and phosphorylated 159-175 peptide. The peptide sequence is reported in the figure with its interpretation; the asterisk indicates the signal corresponding to the phosphorylated tyrosine.

S8. IMAC on BL21(DE3) soluble lysates producing H6-Ph_Sumo-CDKL5(1-352)

To define if the shift of the His tag from the C-terminal to the N-terminal extremity of CDKL5ΔC was sufficient to avoid the contamination of the chromatographic fractions with the N-terminally truncated protein, two different constructs of the target protein were tested: PhSumo-CDKL5(1-352)-His and His-PhSumo-CDKL5(1-352). As shown in Figure S7, both constructs were mainly insoluble in BL21(DE3) lysates (D fractions). However, the soluble protein completely bound the IMAC resin (compare L and FT fractions), and after two wash steps (W1 and W2) it could be collected in the elution fractions (E1 and E2). Regardless of the position of the His-tag, the N-terminally truncated form of CDKL5ΔC was co-eluted with the full-length protein in both cases, suggesting an oligomerization of the two forms of the protein. However, such contamination seems less evident in the preparations of His-Ph_Sumo-CDKL5(1-352).

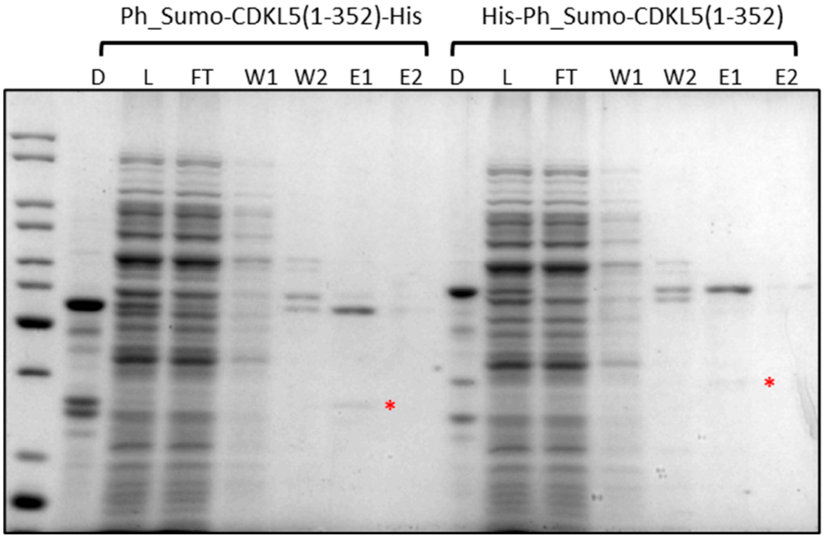

Figure S8 Purification of PhSumo-CDKL5(1-352)-His and His-PhSumo-CDKL5(1-352) from *E. coli* BL21(DE3) soluble lysates. After cellular disruption, the insoluble (D) and soluble (L) fractions were separated by centrifugation. Then the L fraction was loaded onto a HisTrap FF Crude column. The unbound material was collected (FT) and unspecific binders were removed with intermediate wash steps (W1 and W2). Finally, elution was performed in two steps (E1 and E2). The red asterisk highlights the N-terminally truncated form of CDKL5ΔC that was collected in the elution fractions. D fractions were 1:50 diluted before loading onto the SDS-PAGE gel.

S9. Isolation of Hsp60 in absence of soluble CDKL5ΔC

To understand if Hsp60 directly bound the IMAC resin independently from the presence of CDKL5ΔC, we performed two parallel IMACs, one on a lysate containing a partially soluble version

of CDKL5ΔC (Ph_Sumo-CDKL5(1-352)-H8 M10V) and another with a lysate from cells expressing a totally insoluble variant of the target protein (Ec_CDKL5(1-352)-His). Figure S8 shows that soluble Ph_Sumo-CDKL5(1-352)-H8 M10V (L fraction) bound the IMAC resin and after two washes (W1 and W2) was co-eluted with putative Hsp60 (E1 and E2). On the other hand, even if Ec_CDKL5(1-352)-His was totally insoluble (D and L fractions), Hsp60 could still be purified with the IMAC resin (E1 and E2 fractions). This suggests that Hsp60 can directly bind the IMAC resin in our experimental setup.

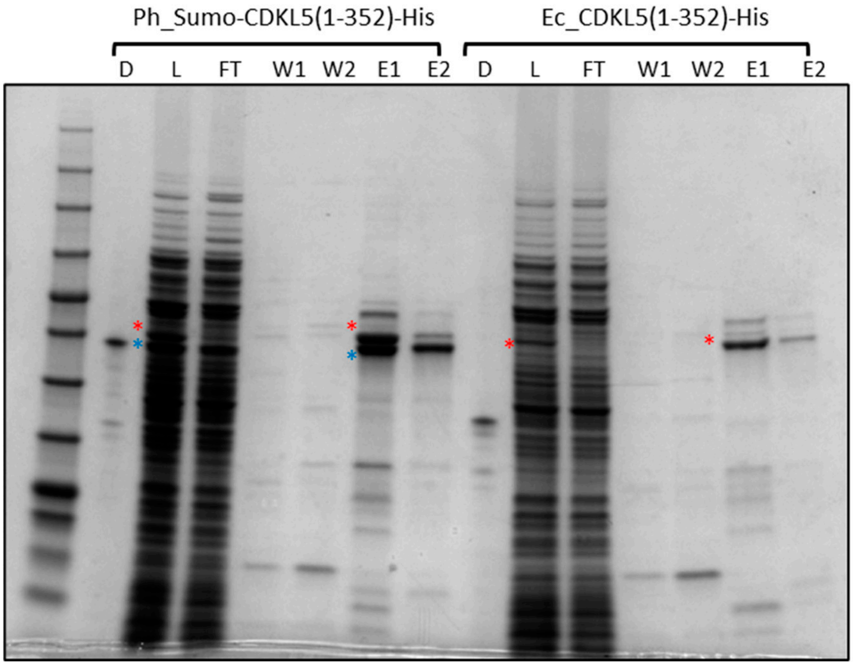

Figure S9 Purification of PhSumo-CDKL5(1-352)-His M10V and Ec_CDKL5(1-352)-H6 from *E. coli* BL21(DE3) soluble lysates. After cellular disruption, the insoluble (D) and soluble (L) fractions were separated by centrifugation. Then the L fraction was loaded onto a Ni Sepharose 6 Fast Flow resin column. The unbound material was collected (FT) and unspecific binders were removed with intermediate wash steps (W1 and W2). Finally, elution was performed in two steps (E1 and E2). The red asterisks highlight putative Hsp60, while the blue asterisks indicate PhSumo-CDKL5(1-352)-His M10V. D fractions were 1:50 diluted before loading onto the SDS-PAGE gel.

Table S1. Composition of buffers used to solubilize CDKL5ΔC Ibs.

A: 50 mM sodium phosphate pH 12.0, 1 mM DTT
B: 50 mM sodium phosphate pH 12.0, 2 M Urea, 1 mM DTT
C: 50 mM sodium phosphate pH 12.0, 0.4 M NaCl, 1 mM DTT
D: 50 mM sodium phosphate pH 12.0, 0.4 M NaCl, 2 M Urea, 1 mM DTT
E: 30 mM sodium phosphate pH 7.0, 0.2% N-lauroylsarcosine (NLS), 1 mM DTT

Table S2. List of primers used in this work.

Primer name	Sequence (5' – 3')	Purpose
EcTat11CDKL5_NdeI_fw	CATCATATGGGTTATGGACGTAAGAAGCGT	Amplification of 5' of EcCDKL5 for cloning into pET40b
EcCDKL5dC_XhoI_rv	AAAACTCGAGGCCCACGCTCAGATTC	Amplification of 3' of EcCDKL5 until aa 352 for cloning into pET40b
PhSumoCDKL5_NdeI_fw	AAACATATGGGTTCGGACTCAGAAGTA	Amplification of 5' of PhSumo for cloning into pET40b
PhCDKL5_NdeI_fw	GTTCATATGGGTAAAATCCCTAACATTGGT	Amplification of 5' of PhCDKL5 for cloning into pET40b
PhCDKL5_303_XhoI_rv	AAAACTCGAGTAAACGTTGTGTCTGAAAAG	Amplification of 3' of PhCDKL5 until aa 303 for cloning into pET40b/pGEX4T1
PhCDKL5_352_XhoI_rv	AAAACTCGAGGCCAACAGAAAGATTCT	Amplification of 3' of PhCDKL5 until aa 352 for cloning into pET40b/pGEX4T1
PhCDKL5_498_XhoI_rv	AAAACTCGAGTACACTCTTAGAGTCG	Amplification of 3' of PhCDKL5 until aa 498 for cloning into pET40b/pGEX4T1
PhCDKL5_2_BamHI_fw	AAAGGATCCAAAATCCCTAACATTGGTA	Amplification of 5' of PhCDKL5 for cloning into pGEX4T1
PhCDKL5_V10M_fw	CCCTAACATTGGTAATGTAATGAACAAATTCGAAATTCTTGGTG	Reversion of M10 in CDKL5
PhCDKL5_V10M_rv	CACCAAGAATTTCGAATTTGTTCATTACATTACCAATGTTAGGG	Reversion of M10 in CDKL5
PhCDKL5_G7_GGT_GGG_wt	GGCAGCAAAATCCCTAACATTGGGAATGTAATGAACAAATTCGAAAT	Introduction of a silent mutation at the level of G7 in the CDKL5 sequence
PhCDKL5_G7_GGT_GGG_rv	ATTTCGAATTTGTTCATTACATTCCCAATGTTAGGGATTTTGCTGCC	Introduction of a silent mutation at the level of G7 in the CDKL5 sequence

References

1. Muñoz IM, Morgan ME, Peltier J, Weiland F, Gregorczyk M, Brown FC, et al. Phosphoproteomic screening identifies physiological substrates of the CDKL 5 kinase. EMBO J. 2018;37(24):1–19.

2. Kameshita I, Sekiguchi M, Hamasaki D, Sugiyama Y, Hatano N, Suetake I, et al. Cyclin-dependent kinase-like 5 binds and phosphorylates DNA methyltransferase 1. Biochem Biophys Res Commun. 2008;377(4):1162–7.

3. Baltussen LL, Negraes PD, Silvestre M, Claxton S, Moeskops M, Christodoulou E, et al. Chemical genetic identification of CDKL 5 substrates reveals its role in neuronal microtubule dynamics. EMBO J. 2018;37(24):1–18.

4. Federico A, Sepe R, Cozzolino F, Piccolo C, Iannone C, Iacobucci I, et al. The complex CBX7-PRMT1 has a critical role in regulating E-cadherin gene expression and cell migration. Biochim Biophys acta Gene Regul Mech. 2019 Apr;1862(4):509–21.

5. Cozzolino F, Iacobucci I, Monaco V, Angrisano T, Monti M. Lysines Acetylome and Methylome Profiling of H3 and H4 Histones in Trichostatin A-Treated Stem Cells. Int J Mol Sci. 2021 Feb;22(4).
